# Erratum

**DOI:** 10.1111/jch.14346

**Published:** 2021-11-21

In the article (J Clin Hypertens (Greenwich). 2021;23(3):595‐605. doi: 10.1111/jch.14113), some unfortunate errors occurred and the authors would like to correct the errors as written below. Specifically, the authors miscalculated the net reclassification improvement (NRI) which needs correction. Although there is a large change in the NRI, the authors believe it does not significantly affect the conclusion of the study as other data supports the conclusion. However, the authors apologize to the readers for the mistakes.

1. In page 596 (line 8‐12), abstract line 16‐20

**Before correction**

The office blood pressure (BP) threshold of 130/80 mmHg was better able to diagnose uncontrolled out‐of‐office BP than 140/90mmHg, and the NRI was 0.255. The automated office BP (AOBP) threshold of 130/80 mmHg also revealed better diagnostic accuracy than 140/90, with NRI of 0.543.

**After correction**

The office BP threshold of 130/80 mmHg was better able to diagnose uncontrolled out‐of‐office BP than 140/90 mmHg (accuracy 74.1% vs 60.9%) although the NRI was ‐0.044. The AOBP threshold of 130/80 mmHg also revealed better diagnostic accuracy than 140/90 mmHg (accuracy 73.1% vs 54.1%), with NRI of 0.045.

2. In page 598 2^nd^ to last line to page 599 line 1

**Before correction**

The threshold of 130/80 mmHg was better able to diagnose uncontrolled out of office BP than was a threshold of 140/90 mmHg, and the NRI was 0.255.

**After correction**

Overall, the threshold of 130/80 mmHg was better able to diagnose uncontrolled out of office BP than was a threshold of 140/90 mmHg (accuracy 74.1% vs 60.9%), although the NRI was ‐0.044.

3. In page 599 line 6‐10

**Before correction**

The AOBP of 130/80 mmHg revealed better diagnostic accuracy than the AOBP of 140/90 mmHg; the NRI was 0.543. The accuracy of AOBP threshold of 130/80 mmHg was higher than that of office BP threshold of 140/90 mmHg (73.1% vs. 54.1%; Table S3 and Table S4).

**After correction**

The AOBP of 130/80 mmHg revealed better diagnostic accuracy than the AOBP of 140/90 mmHg. The accuracy of AOBP threshold of 130/80 mmHg was higher than that of office BP threshold of 140/90 mmHg (73.1% vs 54.1%, Table S3 and Table S4), with the NRI of 0.045.

4. In page 599, line 12‐13.

**Before correction**

accuracy for identifying uncontrolled out‐of‐office BP (Table 4, NRI = 0.076).

**After correction**

accuracy for identifying uncontrolled out‐of‐office BP (Table 4, NRI = 0.060).

5. In page 601, 8th to 5th line from bottom

**Before correction**

office BP threshold of 130/80 mmHg showed better accuracy than 140/90 mmHg (Table S5, Table S6, Table S7, Table S8). The NRIs of office BP and AOBP thresholds of 130/80 mmHg were 0.461 and 0.490, respectively (Table S9).

**After correction**

office BP threshold of 130/80 mmHg showed better accuracy than 140/90 mmHg (Table S5, Table S6, Table S7, Table S8), although the NRIs of office BP and AOBP thresholds of 130/80 mmHg were ‐0.085 and 0.069, respectively (Table S9).

6. In page 601, Table 3

**Before correction**

**Table:** 

Daytime BP 135/85 mmHgorHome BP 135/85 mmHg	Office BP 140/90 mmHg	Office BP 130/80 mmHg	Reclassification improvement	Automated office BP 140/90 mmHg	Automated office BP 130/80 mmHg	Reclassification improvement
Controlled (N=111)	Controlled (N=79)^a^	Controlled (N=44)	‐0.152^i^	Controlled (N=89)^k^	Controlled (N=56)	‐0.113^s^
		Uncontrolled (N=35)^e^			Uncontrolled (N=33)^o^	
	Uncontrolled (N=32)^b^	Controlled (N=0)^f^		Uncontrolled (N=22)^l^	Controlled (N=0)^p^	
		Uncontrolled (N=32)			Uncontrolled (N=22)	
Uncontrolled (N=357)	Controlled (N=151)^c^	Controlled (N=54)	0.407^j^	Controlled (N=193)^m^	Controlled (N=71)	0.656^t^
		Uncontrolled (N=97)^g^			Uncontrolled (N=122)^q^	
	Uncontrolled (N=206)^d^	Controlled (N=0)^h^		Uncontrolled (N=164)^n^	Controlled (N=0)^r^	
		Uncontrolled (N=206)			Uncontrolled (N=164)	
i=(f‐e)/(a+c); j=(g‐h)/(b+d); Net reclassification improvement = i+j = 0.255. s=(p‐o)/(k+m); t=(q‐r)/(l+n); Net reclassification improvement = s+t = 0.543.						

**After correction**

**Table:** 

Daytime BP 135/85 mmHgorHome BP 135/85 mmHg	Office BP 140/90 mmHg	Office BP 130/80 mmHg	Reclassification improvement	Automated office BP 140/90 mmHg	Automated office BP 130/80 mmHg	Reclassification improvement
Controlled (N=111)^a^	Controlled (N=79)	Controlled (N=44)	‐0.315^g^	Controlled (N=89)	Controlled (N=56)	‐0.297^m^
		Uncontrolled (N=35)^c^			Uncontrolled (N=33)^i^	
	Uncontrolled (N=32)	Controlled (N=0)^d^		Uncontrolled (N=22)	Controlled (N=0)^j^	
		Uncontrolled (N=32)			Uncontrolled (N=22)	
Uncontrolled (N=357)^b^	Controlled (N=151)	Controlled (N=54)	0.272^h^	Controlled (N=193)	Controlled (N=71)	0.341^n^
		Uncontrolled (N=97)^e^			Uncontrolled (N=122)^k^	
	Uncontrolled (N=206)	Controlled (N=0)^f^		Uncontrolled (N=164)	Controlled (N=0)^l^	
		Uncontrolled (N=206)			Uncontrolled (N=164)	
g=(d‐c)/a; h=(e‐f)/b; Net reclassification improvement = g+h = ‐0.044. m=(j‐i)/a; n=(k‐l)/b; Net reclassification improvement = m+n = 0.045.						

7. In page 602, Table 4

**Before correction**

**Table:** 

Daytime BP 135/85 mmHgorHome BP 135/85 mmHg	Office BP 130/80 mmHg	Automated office BP 130/80 mmHg	Reclassification improvement
Controlled (N=111)	Controlled (N=44)^a^	Controlled (N=40)	0.122^i^
		Uncontrolled (N=4)^e^	
	Uncontrolled (N=67)^b^	Controlled (N=16)^f^	
		Uncontrolled (N=51)	
Uncontrolled (N=357)	Controlled (N=54)^c^	Controlled (N=35)	‐0.046^j^
		Uncontrolled (N=19)^g^	
	Uncontrolled (N=303)^d^	Controlled (N=36)^h^	
		Uncontrolled (N=267)	
i=(f‐e)/(a+c); j=(g‐h)/(b+d) NRI=i+j=0.076.			

**After correction**

**Table:** 

Daytime BP 135/85 mmHgorHome BP 135/85 mmHg	Office BP 130/80 mmHg	Automated office BP 130/80 mmHg	Reclassification improvement
Controlled (N=111)^a^	Controlled (N=44)	Controlled (N=40)	0.108^g^
		Uncontrolled (N=4)^c^	
	Uncontrolled (N=67)	Controlled (N=16)^d^	
		Uncontrolled (N=51)	
Uncontrolled (N=357)^b^	Controlled (N=54)	Controlled (N=35)	‐0.048^h^
		Uncontrolled (N=19)^e^	
	Uncontrolled (N=303)	Controlled (N=36)^f^	
		Uncontrolled (N=267)	
g=(d‐c)/a; h=(e‐f)/b NRI=g+h=0.060.			

8. In supplemental material, Supplemental Table 1

**Before correction**

**Table:** 

		**Out‐of‐office BP**		
		Controlled	Uncontrolled	Subtotal
Office BP	Controlled	79	151	230
	Uncontrolled	32	206	
	Subtotal	111	357	468
Sensitivity		71.2%		
Specificity		57.7%		
Accuracy		60.9%		
Positive predictive value		34.3%		
Negative predictive value		86.6%		

**After correction**

**Table:** 

		**Out‐of‐office BP**		
		Controlled	Uncontrolled	Subtotal
Office BP	Controlled	79	151	230
	Uncontrolled	32	206	238
	Subtotal	111	357	468
Sensitivity		71.2%		
Specificity		57.7%		
Accuracy		60.9%		
Positive predictive value		34.3%		
Negative predictive value		86.6%		

9. In supplemental material, Supplemental Table 2

**Before correction**

**Table:** 

		**Out‐of‐office BP**		
		Controlled	Uncontrolled	Subtotal
Office BP	Controlled	44	54	230
	Uncontrolled	67	303	
	Subtotal	111	357	468
Sensitivity		39.6%		
Specificity		84.9%		
Accuracy		74.1%		
Positive predictive value		44.9%		
Negative predictive value		81.9%		

**After correction**

**Table:** 

		**Out‐of‐office BP**		
		Controlled	Uncontrolled	Subtotal
Office BP	Controlled	44	54	98
	Uncontrolled	67	303	370
	Subtotal	111	357	468
Sensitivity		39.6%		
Specificity		84.9%		
Accuracy		74.1%		
Positive predictive value		44.9%		
Negative predictive value		81.9%		

10. In supplemental material, Supplemental Table 9

**Before correction**

**Supplemental Table** 9. **Reclassification tables for uncontrolled out‐of‐office BP (daytime BP or home BP≥130/80 mmHg) according to change of office BP threshold from 140/90 mmHg to 135/85 mmHg and according to change of automated office BP threshold from 140/90 mmHg to 135/85 mmHg**

**Table:** 

Daytime BP 130/80 mmHgorHome BP 130/80 mmHg	Office BP 140/90 mmHg	Office BP 130/80 mmHg	Reclassification improvement	Automated office BP 140/90 mmHg	Automated office BP 130/80 mmHg	Reclassification improvement
Controlled (N=59)	Controlled (N=44)^a^	Controlled (N=23)	‐0.091^i^	Controlled (N=48)^k^	Controlled (N=32)	‐0.057^s^
		Uncontrolled (N=21)^e^			Uncontrolled (N=16)^o^	
	Uncontrolled (N=15)^b^	Controlled (N=0)^f^		Uncontrolled (N=11)^l^	Controlled (N=0)^p^	
		Uncontrolled (N=15)			Uncontrolled (N=11)	
Uncontrolled (N=409)	Controlled (N=186)^c^	Controlled (N=75)	0.552^j^	Controlled (N=234)^m^	Controlled (N=95)	0.747^t^
		Uncontrolled (N=111)^g^			Uncontrolled (N=139)^q^	
	Uncontrolled (N=223)^d^	Controlled (N=0)^h^		Uncontrolled (N=175)^n^	Controlled (N=0)^r^	
		Uncontrolled (N=223)			Uncontrolled (N=175)	
i=(f‐e)/(a+c); j=(g‐h)/(b+d); Net reclassification improvement = i+j = 0.461. s=(p‐o)/(k+m); t=(q‐r)/(l+n); Net reclassification improvement = s+t = 0.490.						

**After correction**

**Supplemental Table** 9. **Reclassification tables for uncontrolled out‐of‐office BP (daytime BP or home BP≥130/80 mmHg) according to change of office BP threshold from 140/90 mmHg to 130/80 mmHg and according to change of automated office BP threshold from 140/90 mmHg to 130/80 mmHg**

**Table:** 

Daytime BP 130/80 mmHgorHome BP 130/80 mmHg	Office BP 140/90 mmHg	Office BP 130/80 mmHg	Reclassification improvement	Automated office BP 140/90 mmHg	Automated office BP 130/80 mmHg	Reclassification improvement
Controlled (N=59)^a^	Controlled (N=44)	Controlled (N=23)	‐0.356^g^	Controlled (N=48)	Controlled (N=32)	‐0.271^m^
		Uncontrolled (N=21)^c^			Uncontrolled (N=16)^i^	
	Uncontrolled (N=15)	Controlled (N=0)^d^		Uncontrolled (N=11)	Controlled (N=0)^j^	
		Uncontrolled (N=15)			Uncontrolled (N=11)	
Uncontrolled (N=409)^b^	Controlled (N=186)	Controlled (N=75)	0.271^h^	Controlled (N=234)	Controlled (N=95)	0.340^n^
		Uncontrolled (N=111)^e^			Uncontrolled (N=139)^k^	
	Uncontrolled (N=223)	Controlled (N=0)^f^		Uncontrolled (N=175)	Controlled (N=0)^l^	
		Uncontrolled (N=223)			Uncontrolled (N=175)	
g=(d‐c)/a; h=(e‐f)/b; Net reclassification improvement = g+h = ‐0.085. m=(j‐i)/a; n=(k‐l)/b; Net reclassification improvement = m+n = 0.069.						

## Supporting information



Supporting

