# Supplementary material for: Erratum
Source: J Clin Hypertens (Greenwich). 2021 Nov 21;24(1):91–6. doi: 10.1111/jch.14346 (PMC8783320; doi:10.1111/jch.14346)

**Supplemental Table 1. Cross-tabulation of office BP threshold of 140/90 mmHg and out-of-office BP threshold of 135/85 mmHg**

|  |  | **Out-of-office BP** | |  |
| --- | --- | --- | --- | --- |
|  |  | Controlled | Uncontrolled | Subtotal |
| Office BP | Controlled | 79 | 151 | 230 |
|  | Uncontrolled | 32 | 206 | 238 |
|  | Subtotal | 111 | 357 | 468 |
| Sensitivity | | 71.2% |  |  |
| Specificity | | 57.7% |  |  |
| Accuracy | | 60.9% |  |  |
| Positive predictive value | | 34.3% |  |  |
| Negative predictive value | | 86.6% |  |  |

**Supplemental Table 2. Cross-tabulation of office BP threshold of 130/80 mmHg and out-of-office BP threshold of 135/85 mmHg**

|  |  | **Out-of-office BP** | |  |
| --- | --- | --- | --- | --- |
|  |  | Controlled | Uncontrolled | Subtotal |
| Office BP | Controlled | 44 | 54 | 98 |
|  | Uncontrolled | 67 | 303 | 370 |
|  | Subtotal | 111 | 357 | 468 |
| Sensitivity | | 39.6% |  |  |
| Specificity | | 84.9% |  |  |
| Accuracy | | 74.1% |  |  |
| Positive predictive value | | 44.9% |  |  |
| Negative predictive value | | 81.9% |  |  |

**Supplemental Table 3. Cross-tabulation of automated office BP threshold of 140/90 mmHg and out-of-office BP threshold of 135/85 mmHg**

|  |  | **Out-of-office BP** | |  |
| --- | --- | --- | --- | --- |
|  |  | Controlled | Uncontrolled | Subtotal |
| Automated Office BP | Controlled | 89 | 193 | 127 |
|  | Uncontrolled | 22 | 164 | 341 |
|  | Subtotal | 111 | 357 | 468 |
| Sensitivity | | 80.2% |  |  |
| Specificity | | 45.9% |  |  |
| Accuracy | | 54.1% |  |  |
| Positive predictive value | | 31.6% |  |  |
| Negative predictive value | | 88.2% |  |  |

**Supplemental Table 4. Cross-tabulation of automated office BP threshold of 130/80 mmHg and out-of-office BP threshold of 135/85 mmHg**

|  |  | **Out-of-office BP** | |  |
| --- | --- | --- | --- | --- |
|  |  | Controlled | Uncontrolled | Subtotal |
| Automated Office BP | Controlled | 56 | 71 | 127 |
|  | Uncontrolled | 55 | 286 | 341 |
|  | Subtotal | 111 | 357 | 468 |
| Sensitivity | | 50.5% |  |  |
| Specificity | | 80.1% |  |  |
| Accuracy | | 73.1% |  |  |
| Positive predictive value | | 44.1% |  |  |
| Negative predictive value | | 83.9% |  |  |

**Supplemental Table 5. Cross-tabulation of office BP threshold of 140/90 mmHg and out-of-office BP threshold of 130/80 mmHg**

|  |  | **Out-of-office BP** | |  |
| --- | --- | --- | --- | --- |
|  |  | Controlled | Uncontrolled | Subtotal |
| Office BP | Controlled | 44 | 186 | 230 |
|  | Uncontrolled | 15 | 223 | 238 |
|  | Subtotal | 59 | 411 | 468 |
| Sensitivity | | 74.6% |  |  |
| Specificity | | 54.5% |  |  |
| Accuracy | | 57.1% |  |  |
| Positive predictive value | | 19.1% |  |  |
| Negative predictive value | | 93.7% |  |  |

**Supplemental Table 6. Cross-tabulation of office BP threshold of 130/80 mmHg and out-of-office BP threshold of 130/80 mmHg**

|  |  | **Out-of-office BP** | |  |
| --- | --- | --- | --- | --- |
|  |  | Controlled | Uncontrolled | Subtotal |
| Office BP | Controlled | 23 | 75 | 98 |
|  | Uncontrolled | 36 | 334 | 370 |
|  | Subtotal | 59 | 441 | 468 |
| Sensitivity | | 38.9% |  |  |
| Specificity | | 81.6% |  |  |
| Accuracy | | 76.3% |  |  |
| Positive predictive value | | 23.5% |  |  |
| Negative predictive value | | 90.3% |  |  |

**Supplemental Table 7. Cross-tabulation of automated office BP threshold of 140/90 mmHg and out-of-office BP threshold of 130/80 mmHg**

|  |  | **Out-of-office BP** | |  |
| --- | --- | --- | --- | --- |
|  |  | Controlled | Uncontrolled | Subtotal |
| Automated Office BP | Controlled | 48 | 234 | 282 |
|  | Uncontrolled | 11 | 175 | 186 |
|  | Subtotal | 59 | 441 | 468 |
| Sensitivity | | 81.4% |  |  |
| Specificity | | 42.8% |  |  |
| Accuracy | | 47.7% |  |  |
| Positive predictive value | | 17.0% |  |  |
| Negative predictive value | | 94.1% |  |  |

**Supplemental Table 8. Cross-tabulation of automated office BP threshold of 130/80 mmHg and out-of-office BP threshold of 130/80 mmHg**

|  |  | **Out-of-office BP** | |  |
| --- | --- | --- | --- | --- |
|  |  | Controlled | Uncontrolled | Subtotal |
| Automated Office BP | Controlled | 32 | 95 | 127 |
|  | Uncontrolled | 27 | 314 | 341 |
|  | Subtotal | 59 | 441 | 468 |
| Sensitivity | | 54.2% |  |  |
| Specificity | | 76.8% |  |  |
| Accuracy | | 73.9% |  |  |
| Positive predictive value | | 25.2% |  |  |
| Negative predictive value | | 92.1% |  |  |

**Supplemental Table 9. Reclassification tables for uncontrolled out-of-office BP (daytime BP or home BP≥130/80 mmHg) according to change of office BP threshold from 140/90 mmHg to 130/80 mmHg and according to change of automated office BP threshold from 140/90 mmHg to 130/80 mmHg**

| Daytime BP 130/80 mmHg  or  Home BP 130/80 mmHg | Office BP 140/90 mmHg | Office BP 130/80 mmHg | Reclassification improvement | Automated office BP 140/90 mmHg | Automated office BP 130/80 mmHg | Reclassification improvement | |
| --- | --- | --- | --- | --- | --- | --- | --- |
| Controlled (N=59)^a^ | Controlled (N=44) | Controlled (N=23) | -0.356^g^ | Controlled (N=48) | Controlled (N=32) | -0.271^m^ | |
|  |  | Uncontrolled (N=21)^c^ |  |  | Uncontrolled (N=16)^i^ |  |  |
|  | Uncontrolled (N=15) | Controlled (N=0)^d^ |  | Uncontrolled (N=11) | Controlled (N=0)^j^ |  |  |
|  |  | Uncontrolled (N=15) |  |  | Uncontrolled (N=11) |  |  |
| Uncontrolled (N=409)^b^ | Controlled (N=186) | Controlled (N=75) | 0.271^h^ | Controlled (N=234) | Controlled (N=95) | 0.340^n^ | |
|  |  | Uncontrolled (N=111)^e^ |  |  | Uncontrolled (N=139)^k^ |  |  |
|  | Uncontrolled (N=223) | Controlled (N=0)^f^ |  | Uncontrolled (N=175) | Controlled (N=0)^l^ |  |  |
|  |  | Uncontrolled (N=223) |  |  | Uncontrolled (N=175) |  |  |
| g=(d-c)/a; h=(e-f)/b; Net reclassification improvement = g+h = -0.085.  m=(j-i)/a; n=(k-l)/b; Net reclassification improvement = m+n = 0.069. | | | | | | |  |

**Supplemental figure 1. Scatter plots of office BP (A) and automated office BP (B) of total subjects according to out-of-office BP control status.**


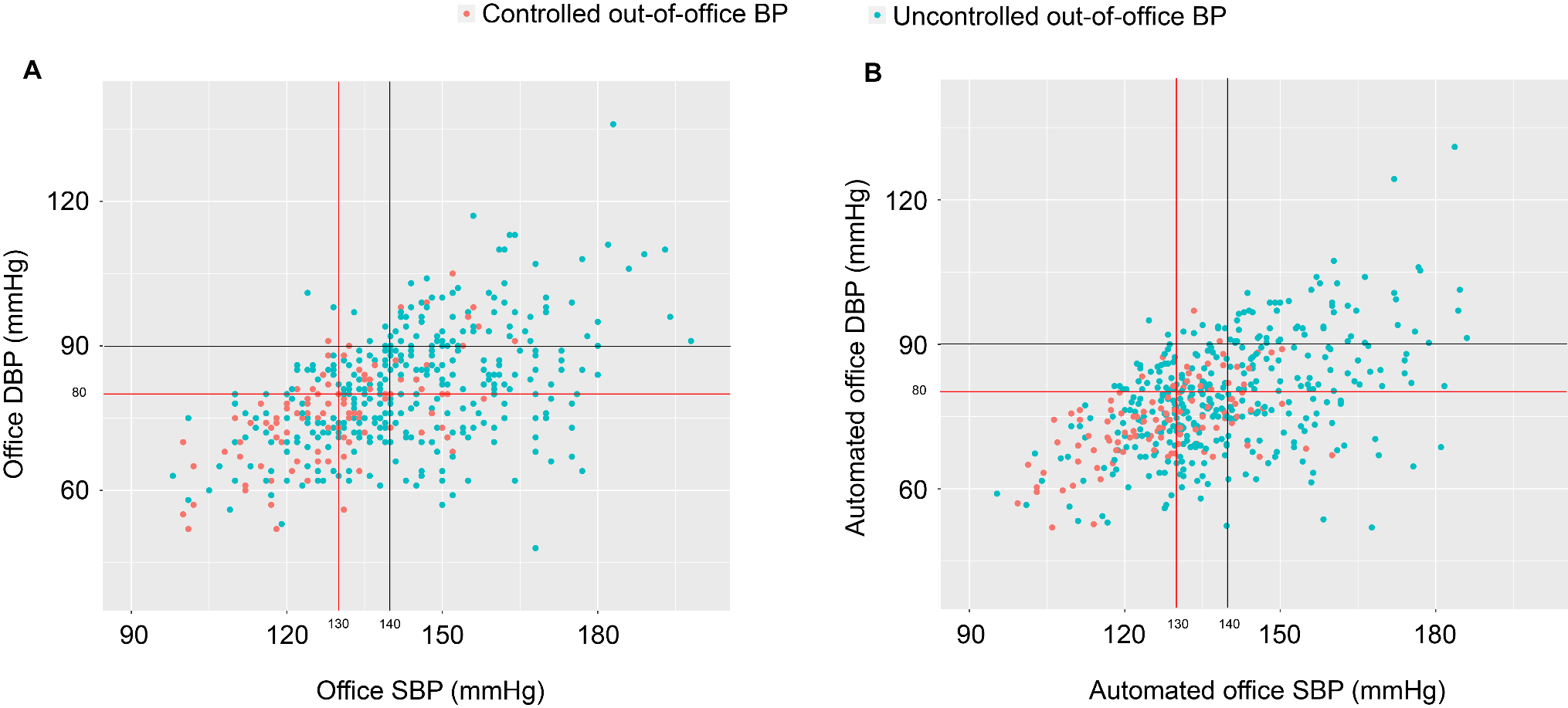

Supplement: Supplementary file 1 — Supporting [file JCH-24-91-s001.docx]
